# Post-Hypoxic Recovery of Respiratory Rhythm Generation Is Gender Dependent

**DOI:** 10.1371/journal.pone.0060695

**Published:** 2013-04-08

**Authors:** Alfredo J. Garcia, Naama Rotem-Kohavi, Atsushi Doi, Jan-Marino Ramirez

**Affiliations:** 1 Center for Integrative Brain Research, Seattle Children’s Research Institute, Seattle, Washington, United States of America; 2 Department of Neurological Surgery, University of Washington, Seattle, Washington, United States of America; University of Cincinnatti, United States of America

## Abstract

The preBötzinger complex (preBötC) is a critical neuronal network for the generation of breathing. Lesioning the preBötC abolishes respiration, while when isolated in vitro, the preBötC continues to generate respiratory rhythmic activity. Although several factors influence rhythmogenesis from this network, little is known about how gender may affect preBötC function. This study examines the influence of gender on respiratory activity and in vitro rhythmogenesis from the preBötC. Recordings of respiratory activity from neonatal mice (P10–13) show that sustained post-hypoxic depression occurs with greater frequency in males compared to females. Moreover, extracellular population recordings from the preBötC in neonatal brainstem slices (P10–13) reveal that the time to the first inspiratory burst following reoxygenation (TTFB) is significantly delayed in male rhythmogenesis when compared to the female rhythms. Altering activity of ATP sensitive potassium channels (K_ATP_) with either the agonist, diazoxide, or the antagonist, tolbutamide, eliminates differences in TTFB. By contrast, glucose supplementation improves post-hypoxic recovery of female but not male rhythmogenesis. We conclude that post-hypoxic recovery of respiration is gender dependent, which is, in part, centrally manifested at the level of the preBötC. Moreover, these findings provide potential insight into the basis of increased male vulnerability in a variety of conditions such as Sudden Infant Death Syndrome (SIDS).

## Introduction

Several pathophysiological conditions have gender-dependent outcomes. For example, females recover better from traumatic brain injury, ischemia and trauma; whereas, males show greater neuronal cell loss and lesion size, which likely contributes to a greater male mortality [1]. The incidence of obstructive sleep apnea is three times greater in men than in women [2]. Similarly in children, respiratory related disorders are also significantly increased in boys [3] [4]. Adult models of sleep-disordered breathing reveal that males exhibit greater impaired wakefulness and co-incidental increases in oxidative stress [5]. In SIDS, approximately 60% of the children succumbing to the syndrome are boys [6], [7], [8]. A common event in traumatic brain injury, sleep apnea and SIDS is the occurrence of hypoxic insult to the central nervous system (CNS) followed by reoxygenation. However, gender differences observed in these conditions may not occur during hypoxia itself, but rather manifest in the acute post-hypoxic recovery. While much attention has been focused on the hypoxic respiratory response [9], the post-hypoxic recovery of respiration is comparatively understudied. This is surprising given that failure to autoresuscitate during transient periods of hypoxia is implicated in the etiology of SIDS [10], [11], [12]. Respiratory dysfunction often involves altered function in the autonomic nervous system, yet again, little is known about the influence gender may have on the function of central networks controlling respiration. We investigated the gender influence on acute post-hypoxic recovery both in vivo and in the preBötzinger complex (preBötC), a neuronal network critically involved in respiratory control.

The preBötC is located within the ventrolateral medulla and is essential to the generation of normal breathing, sighing and gasping [13], [14], [15], [16]. Pathological disturbances of the preBötC are associated with morbidity and mortality in humans [17], and individuals with bilateral medullary lesions encompassing preBotC fail to breathe [18]. Rhythmic neuronal activity from the preBötC can be preserved in vitro where the neuronal rhythm responds to hypoxia and reoxygenation in a manner consistent to that observed in vivo [14], [19], [20]. Rhythm generation from the preBötC results from a complex interplay between intrinsic membrane properties of preBötC neurons, synaptic interactions, and neuromodulation [21], but the influence gender may have on the neurophysiology of the preBötC is unknown.

We test the hypothesis that gender differences present in respiration following reoxygenation are preserved at the level of the preBötC. Our in vivo experiments reveal that gender impacts post-hypoxic recovery of ventilation. Similarly, in vitro recordings from the preBötC show that gender influences the time that the respiratory network requires to recover following hypoxia. This is evident when assessing the TTFB. Under both in vivo and in vitro conditions, there is a significantly attenuated post-hypoxic recovery in males. Additionally, in vitro pharmacological experiments implicate a role for metabolic status and K_ATP_ in generating gender differences at the level of the preBötC. Together, these observations suggest that fundamental gender differences in neuronal circuitry are present during the postnatal period and contribute to differences in function.

## Methods

### Ethics Statement

Experiments were conducted using CD1 mice (Postnatal day 2–13) and protocols were approved by Seattle Children’s Research Institute Animal Care and Use Committee in accordance with the National Institutes of Health guidelines.

To minimize artifacts such as movement, mice used for in vivo experimentation were anesthetized with 1.25 to 1.75 mg urethane per gram of the subject delivered via intraperitoneal injection. The final dosage of urethane anesthesia was determined by lack of response to tail pinch. After in vivo experiments were completed, all subjects were euthanized by rapid decapitation. Mice used for tissue harvest of the preBötC were anesthetized with inhaled isoflurane followed by rapid decapitation and isolation of the brainstem. In all cases, postmortem dissection and gonadal identification was used to determine gender.

### Electromyogram

Electromyogram (EMG) recordings were conducted in anesthetized mice (P10–13) of both genders. Recordings were made by placing a teflon-coated silver electrode onto the external intercostal muscles of the subject. Subjects undergoing the EMG protocol were freely breathing and inspired a gas mixture of 95% O_2_ and 5% CO_2_ prior to and following severe hypoxic challenge (inspired gas 95% N_2_ and 5% CO_2_). Baseline metrics were defined as the final 60 sec of control period (i.e., a minimum of 600 sec breathing the control gas) prior to hypoxic exposure. The period of recovery following hypoxia was monitored for a minimum of 300 sec. In many cases, reoxygenation led to a sustained post-hypoxic depression, which was defined as a period >10 sec where post-hypoxic f_inst_ <80% of baseline f_inst_ prior to hypoxia.

The EMG-recorded signal was amplified 10,000X, filtered (low pass, 1.5 kHz; high pass, 250 Hz), rectified, and integrated using an electronic filter. All in vivo recordings were stored on a computer for posthoc analysis.

### Brainstem Slices

Transverse brainstem slices were prepared from both male and female CD1 mice as previously described [22]. Briefly, the isolated brainstem was glued to an agar block (dorsal face to agar) with the rostral face up and submerged into artificial cerebrospinal fluid (aCSF, ∼4°C) equilibrated with carbogen. Serial cuts were made through the brainstem until the appearance of anatomical landmarks such as the hypoglossal nucleus and inferior olive. A single slice (∼600 µm thick) containing the preBötC was cut and taken. The caudal face of the preBötC slice was approximately 0.05 to 0.10 mm rostral from the obex. This slice was retained and transferred into the recording chamber (∼6 mL volume) where it was continuously superfused (12 to 15 mL/min) with recirculating aCSF above and below the slice.

### Media and Pharmacological Agents

The composition of aCSF was (in mM): 118 NaCl, 3.0 KCl, 25 NaHCO_3_, 1 NaH_2_PO_4_, 1.0 MgCl_2_, 1.5 CaCl_2_, 10 D-glucose, 20 Sucrose. Rhythmic activity from the preBötC was induced by raising extracellular KCl to a final concentration of 8.0 mM. In experiments involving 30 mM glucose aCSF, sucrose was replaced with equimolar glucose. All aCSF solutions had an osmolarity of 305 to 312mOSM and a pH of 7.40 to 7.45 when equilibrated with gas mixtures containing 5% CO_2_ at ambient pressure. Control oxygen conditions were made by equilibrating aCSF with carbogen (95% O_2_, 5% CO_2_) while hypoxic conditions were made by aerating with 95% N_2_, 5% CO_2_. Despite the equilibration of aCSF with 0% O_2_, hypoxic media contained some O_2_ [20]. Picrotoxin (PTX), strychnine (STR), tolbutamide (TOL), and diazoxide were obtained from Sigma-Aldrich (St. Louis, MO).

### Population Recordings

In several cases, population recordings from two preBötC brainstem slices were recorded simultaneously within a single recording chamber. In situations where two slices were used, the slices were positioned in a staggered arrangement such that media flow was not obstructed for either preparation. Extracellular population activity was recorded with glass suction pipettes (tip resistance <1 MOhm) filled with aCSF that were positioned over the ventral respiratory column containing the preBötC. The recorded signal was amplified 10,000X, filtered (low pass, 1.5 kHz; high pass, 250 Hz), rectified, and integrated using an electronic filter. Recordings were stored on a computer for posthoc analysis. Rhythm generation originating from the preBötC was recorded in carbogen prior to hypoxia, during hypoxia (95%N_2_ 5%CO_2_), and following hypoxic exposure back to carbogen.

### Media Oxygen Measurements

Oxygen measurements of aCSF were made in the rear of the tissue chamber (>0.2 mm from below the fluid-gas interface) using a custom constructed polarographic oxygen electrode previously described [20], [23]. The electrode was polarized at −700 mV using a polarographic amplifier (AM Systems, Sequim WA).

### Analysis and Statistics

Instantaneous frequency (f_inst_) in EMG recordings was periodically averaged (6 breathes per average). A chi square test was used to test the null hypothesis that the occurrence of post-hypoxic depression was the same between genders. Post hypoxic depression was defined as a period >10 sec where post-hypoxic f_inst_ <80% of baseline f_inst_ prior to hypoxia.

**Figure 1:**
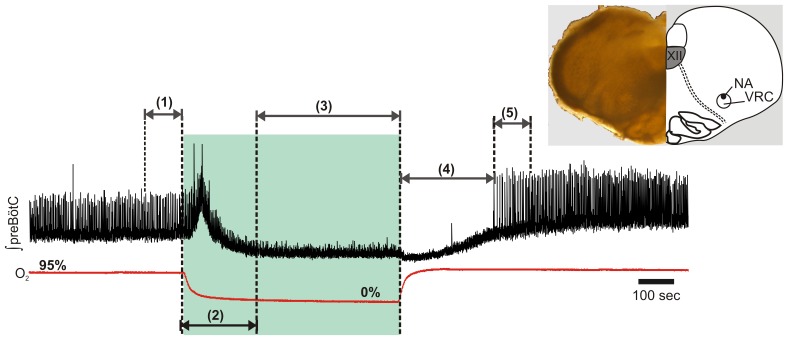
Hypoxia and rexoygenation cause stereotypical changes in rhythmic population activity. Analysis of the in vitro preBötC rhythm was segmented according to neuronal population activity and the state of oxygenation prior to, during, and following hypoxia. (1) steady-state rhythmogenesis in carbogen prior to hypoxia; (2) augmentation during initial hypoxia (0 to 200 sec) (3) steady-state rhythmogenesis during hypoxia (i.e. last 400 sec of hypoxia) (4) TTFB upon reoxygenation; (5) post-hypoxic rhythmogenesis. Inset: The transverse preBötC slice as observed under brightfield low magnification (left). A schematic diagram of the preBötC slice and anatomical landmarks (right). The diagram labels the hypoglossal nucleus (XII), the nucleus ambiguus (NA),and the ventral respiratory column (VRC) containing the preBötC.

The in vitro response to hypoxia and reoxygenation is a well-characterized stereotypical response [20], [24], [25]. Transition from a well-oxygenated state to hypoxia triggers changes in the frequency of rhythmogenesis initially characterized by frequency augmentation and followed by a frequency depression. Upon reoxygenation, the frequency of rhythmogenesis goes through a period of depression that is succeeded by frequency rebound and augmentation. In a recent study, we demonstrated that these stereotypical periods during transitions in oxygenation can be quantitatively described [20]. To thoroughly capture potential gender differences, analysis of rhythmic activity from the in vitro preBötC was segmented into various periods (Figure 1): (1) steady-state rhythmogenesis prior to hypoxia (i.e., the final 100 sec rhythmogenesis in carbogen prior to hypoxia); (2) the hypoxic augmentation of rhythmogenesis (i.e., the initial 200 sec of 600 sec hypoxic exposure); (3) rhythmogenesis during steady-state hypoxia (i.e., the last 400 sec of hypoxia exposure); (4) TTFB upon reoxygenation (i.e., the first regular burst following reoxygenation from hypoxia); (5) post-hypoxic rhythmogenesis (i.e., the first 100 sec following TTFB). Integrated population bursts from extracellular recordings were detected and analyzed post hoc using Clampfit 10. Bursts accepted for analysis during hypoxic augmentation, steady-state hypoxia, and post-hypoxic reoxygenation had amplitudes >25% of the mean steady-state burst amplitude prior to hypoxia for the respective rhythm. The >25% amplitude threshold for burst inclusion was used to ensure that the signal to noise ratio could sufficiently identify a given population burst from background activity.

Kaplan-Meier estimator functions were used to describe the probability of stable rhythmogenesis during hypoxic augmentation phase (i.e., the initial 200 sec of hypoxia). The endpoint for this analysis was defined as the first occurrence of a 10 sec interburst interval during hypoxic augmentation, which was approximately two times greater than the mean interburst interval of rhythmogenesis prior to hypoxia. Rhythms not demonstrating the defined endpoint during hypoxic augmentation were censored. Differences between the Kaplan-Meier estimator functions were determined using the Gehan-Breslow-Wilcoxon test.

Differences between two means were determined by an unpaired t-test. To determine correlations between two metrics, linear regression analysis was performed. In addtion to calculating the r^2^ value, the P-value comparing the regression line slope to 0 was calculated. This P-value determines the probability for a given set of metrics to randomly demonstrate the relationship described by the r^2^ value should those metrics be unrelated. Statistical analyses were conducted using Prism (GraphPad Software Inc., La Jolla, CA), and statistical significance was defined by a P-value ≤0.05. Unless otherwise stated, data plots with error bars represent the mean ± standard error of the mean.

## Results

### Gender Influences Breathing Response Following Severe Hypoxia

**Figure 2:**
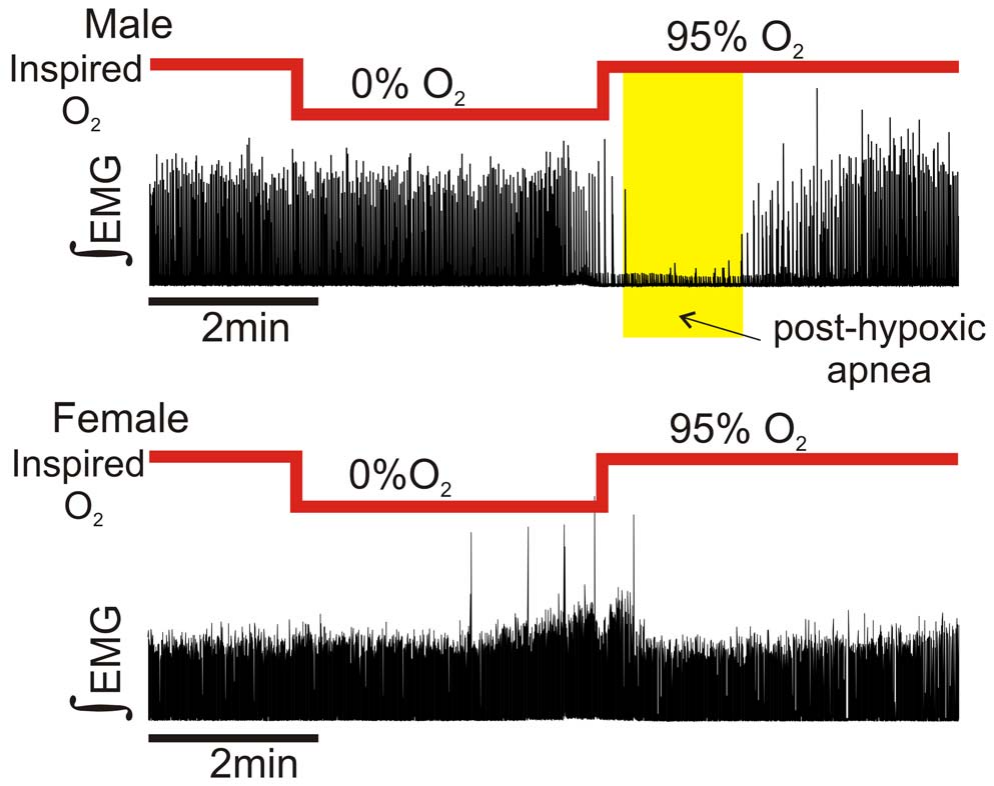
In vivo Gender differences exist in the occurrence of post-hypoxic apnea following exposure to severe hypoxia. Representative traces of integrated electromyogram recordings from both a male (top) and female (bottom) subject. All males (n = 8) exhibited a post-hypoxic apnea following reoxygenation from severe hypoxia (95% N_2_, 5% CO_2_), whereas only 28% of females (n = 3 of 11) exhibited a post-hypoxic apnea.

Prior to hypoxia, respiratory burst frequency in freely breathing mice was 1.14±0.21 Hz (n = 8) and 1.69±0.16 Hz (n = 11) for males and females, respectively. During hypoxia respiratory burst frequency was 1.41±0.22 Hz and 1.73±0.10 Hz for males and females, respectively. No differences were observed in respiratory burst frequency either prior to or during hypoxia. However, reoxygenation from severe hypoxia caused a sustained post-hypoxic depression in all male subjects (n = 8) (Figure 2, upper trace). By contrast, only 27% (n = 3 of 11) of the females exhibited a sustained post-hypoxic depression (Figure 2, lower trace). This gender bias was highly significant (P = 0.003). To determine whether this in vivo gender bias involved differences at the level of the preBötC, an area critical for respiratory rhythm generation in vivo [13], [26], we conducted in vitro slice experiments.

### Central Respiratory Rhythm Generation in Males is Less Reliable during Early Hypoxic Challange and Recovers Later Following Hypoxia

A total of 42 population rhythms from the in vitro preBötC were recorded from gender-identified slices (postnatal days 10–13: n = 19 male, n = 23 female). Similar to the in vivo findings, no gender differences were found in steady-state metrics of rhythmogenesis prior to hypoxia, during hypoxia, or following reoxygenation (Table 1). However, significant gender differences were observed during the transition in oxygen conditions.

**Table 1:** Slice metrics prior to, during, and following hypoxia from preBötC rhythms in gender-identified rhythms.

Metric	Male	Female
n value	19 (8)^1^	23 (11)^1^
Instantaneous Frequency of rhythm prior to hypoxia (Hz)	0.19±0.02	0.22±0.02
Irregularity Score of Period prior to hypoxia	31.31±3.51	29.57±3.53
Instantaneous Frequency of rhythm during hypoxia (Hz)	0.25±0.03	0.24±0.02
Irregularity Score of Period during hypoxia	111.43±35.57	162.79±67.36
Post-hypoxic instantaneous frequency of rhythmogenesis	0.18±0.01	0.19±0.02
Post-hypoxic irregularity score of period of rhythmogenesis	29.96±5.22	25.22±2.40

1 = n values for slices identified with rhythms during steady-state hypoxia. Irregularity Score of period was calculated using the methods described by [49].

**Figure 3:**
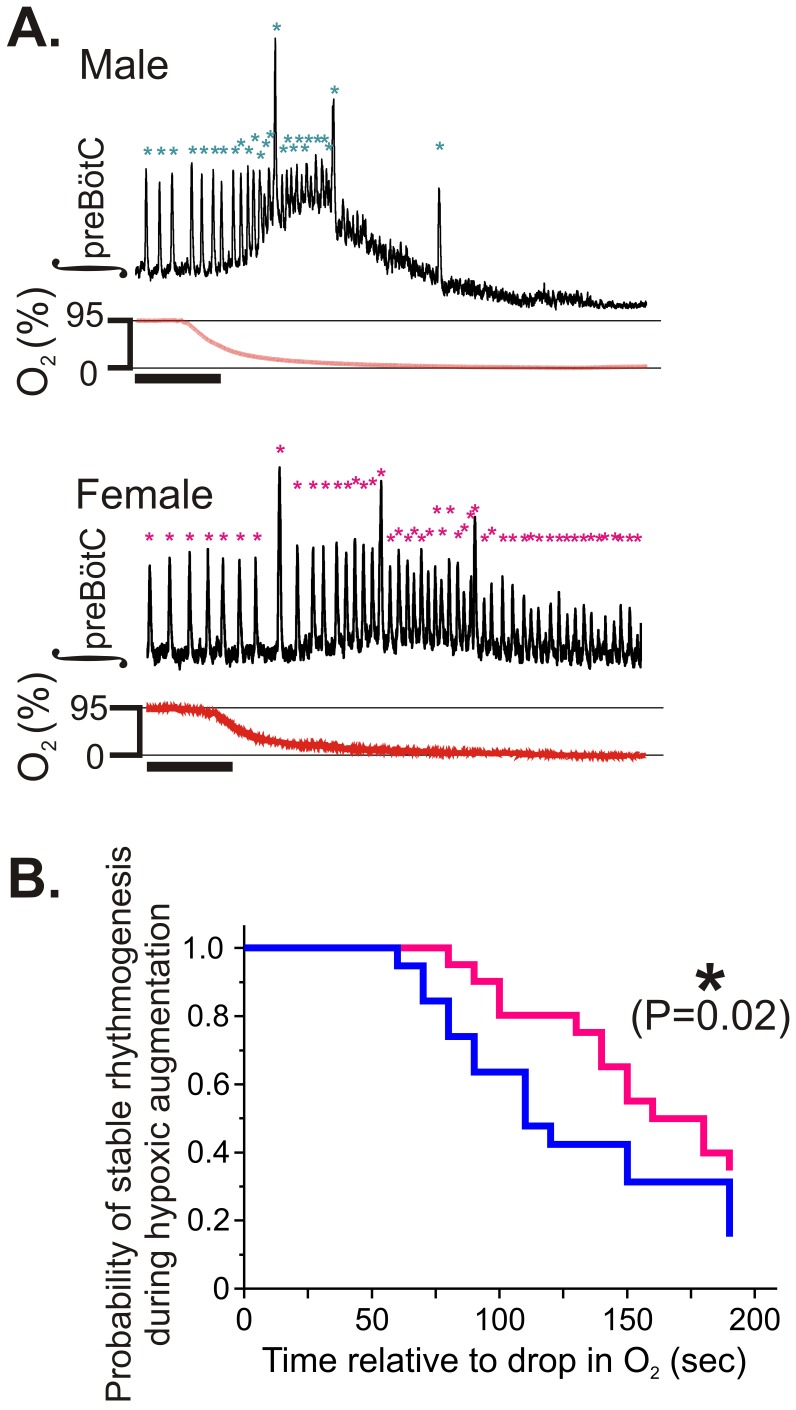
Gender influences rhythm generation during the transition from a well-oxygenated state to hypoxia. (A) Male preBötC rhythms (top) tend to fail more frequently compared to female rhythm (bottom). Asterisks denote detected integrated population bursts and scale bar repesents 20 sec. (B) Kaplan-Meier curves determined for both male (blue) and females (magenta) preBötC rhythms are significantly different from one another. The first 10 sec interburst interval was used as the endpoint metric during the transition to hypoxia.

**Figure 4:**
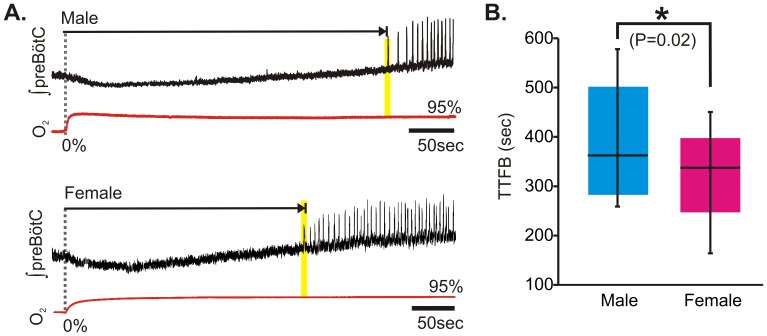
Gender influences the recovery of rhythmogenesis following hypoxia. (A) Representative traces of integrated population activity recovering from hypoxia for both a male (top) and female preBötC slice (bottom) demonstrating the stereotypical post-hypoxic depression in activity. (B) Box-whisker plots comparing TTFB between male (n = 19) and female (n = 23) rhythms.

During hypoxic augmentation, at a time when the frequency of the respiratory rhythm is still elevated compared to baseline, the beginning of a respiratory depression is marked by decrementing burst amplitudes (Figure 3A). During hypoxic augmentation male rhythms failed earlier when compared to female counterparts (Figure 3B; P = 0.02). Similarly, while both genders exhibited a stereotypical pattern of recovery during/following reoxygenation (Figure 4A), there was a quantitative difference in the post-hypoxic recovery of rhythmogenesis. It took significantly longer for males compared to females to generate the first respiratory burst: The TTFB for male slices was 381±21 sec, for females it was 323±13 sec, (P = 0.02; Figure 4B).

### Recovery Following Hypoxia is Frequency Dependent in Males but not Females

**Figure 5:**
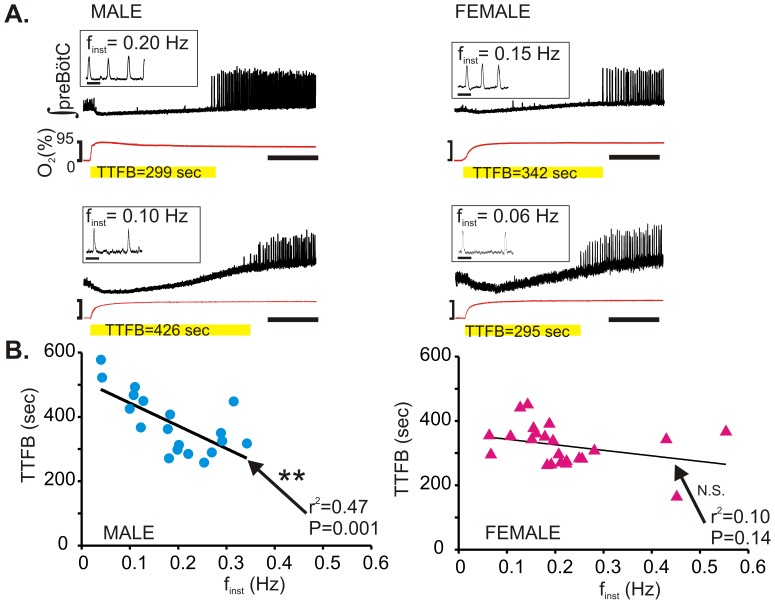
Post-hypoxic recovery of rhythmogenesis correlates to f_inst_ prior to hypoxia in male rhythms but not in female rhythms. (A) Representative traces of post-hypoxic recovery (i.e. TTFB) of male (left) and female (right) rhythms. Scale bar represents 2 min. Insets in each trace is the corresponding rhythm and f_inst_ from each experiment prior to hypoxia; scale bar represents 5 sec. (B) Linear regression analysis of f_inst_ prior to hypoxia to TTFB for both male (left; n = 19) and female (right; n = 23) rhythms. The r^2^ value is greater for the male correlation and possesses a slope significantly different from 0.

We next tested the hypothesis that the instantaneous frequency (f_inst_) of baseline rhythmogenesis prior to hypoxia predicts the timing of the post-hypoxic recovery (i.e. TTFB). The f_inst_ of individual male rhythms was inversely related to TTFB, but no such correlation was found in individual female rhythmic activities (Figure 5A). Linear regression analysis of TTFB to the steady state f_inst_ prior to hypoxia revealed that the regression line of f_inst_ to TTFB of male rhythms (Figure 5B left) had an r^2^ = 0.47 and a slope which was significantly different from 0 (P = 0.001); whereas, in female rhythms the regression line (Figure 5B right) had an r^2^ = 0.10 and a slope that was not different from 0 (P = 0.14). No gender differences were found in the linear regression of TTFB to other rhythmic metrics prior to or during hypoxia (all functions had an r^2^≤0.05 for both genders and/or slopes not significantly different from 0, P≥0.05). Thus, the post-hypoxic recovery was delayed in male slices exhibiting relatively slower rhythms, while the recovery in male slices with comparatively faster rhythms was not different from the recovery of female slices. By contrast, in females the post-hypoxic recovery of respiratory activity was independent of baseline frequency.

### Blockade of K_ATP_ Abolishes the Posthypoxic Gender Difference in Recovery

**Figure 6:**
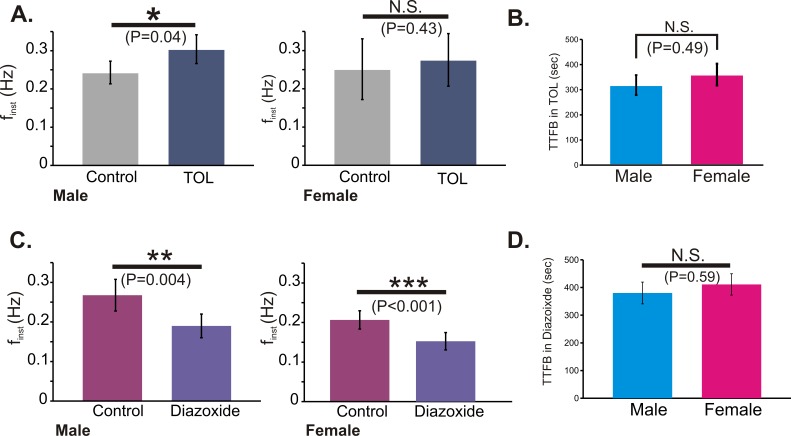
Contribution of K_ATP_ channels on f_inst_ and TTFB. (A) The K_ATP_ channel antagonist, tolbutamide (TOL, 100 to 400 µM), significantly increases f_inst_ of rhythmogenesis from male (n = 9) but not female (n = 9) slices and (B) eliminates the gender difference in TTFB. (C). The K_ATP_ channel agonist, diazoxide (60 to 100 µM), significantly decreases f_inst_ of rhythmogenesis from male (n = 8) and female (n = 9) slices and (D) eliminates the gender difference in TTFB.

It is well established that hypoxia increases the conductance of K_ATP_ which in turn inhibits the frequency of respiratory rhythmic activity [27]. To test whether the K_ATP_ contributed to the gender difference in TTFB, gender identified slices were exposed to hypoxia-reoxygenation in the presence of either the K_ATP_ antagonist, TOL (100 to 400 µM) or the K_ATP_ agonist, diazoxide (60 to 100 µM). TOL application caused a significant increase in f_inst_ of male rhythmic activity prior to hypoxia (P = 0.04) while in females the f_inst_ prior to hypoxia was not significantly affected (Figure 6A; P = 0.43). Moreover, in TOL the TTFB of male rhythms was not significantly different from the TTFB in female rhythms (Figure 6B; P = 0.49 male = 318±40 sec; female = 360±43 sec). Application of diazoxide caused a decrease in the f_inst_ of both male and female rhythms prior to hypoxia (Figure 6C) and eliminated the gender difference in TTFB (Figure 6D; P = 0.59 male = 380±39 sec; female = 411±39 sec). Because the K_ATP_ conductance is regulated by ATP availability and hence, metabolic status, we sought to determine whether increasing glucose availability could also affect the gender difference in post-hypoxic recovery.

**Figure 7:**
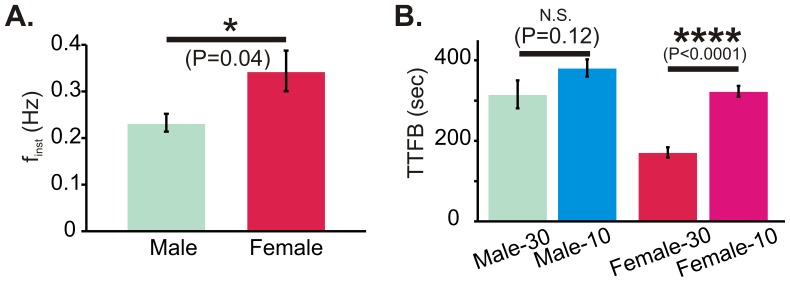
Effect of glucose supplementation on f_inst_ and TTFB. (A) Prior to hypoxia, glucose supplementation (30 mM) causes a significant difference in f_inst_ between male (n = 7) and female (n = 8) rhythms. (B) When compared to TTFB in 10 mM glucose aCSF, glucose supplementation does not affect male TTFB, but significantly reduces female TTFB.

In the brainstem slice preparation, increasing metabolic substrate availability using aCSF containing 30 mM glucose caused female rhythmic activity to be significantly faster (Figure 7A; P = 0.04), and exaggerated the gender biased post-hypoxic recovery. While elevated glucose had no significant effect on the TTFB of male respiratory rhythms (P = 0.12), the TTFB of female rhythmic activity in 30 mM glucose supplemented aCSF was significantly faster compared to the TTFB in aCSF with 10 mM glucose (Figure 7B; P<0.0001).

### Blockade of Synaptic Inhibition does not Alter the Post-hypoxic Gender Difference in Recovery of Respiratory Rhythmic Activity

**Figure 8:**
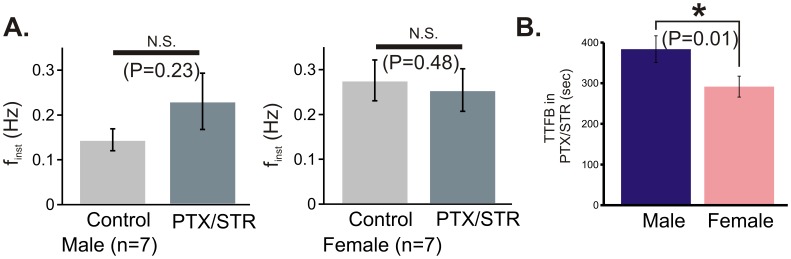
Blockade of fast GABAergic and glycinergic receptors do not prevent gender difference in TTFB. Co-application of the antagonists for fast GABAergic synaptic transmission, picrotoxin (PTX, 50 µM), and the glycineric synaptic transmission, strycnine (STR,1 µM), affects neither (A) f_inst_ prior to hypoxia for either gender nor (B) the gender difference in TTFB.

To test the involvement of synaptic inhibition in mediating the differences in post-hypoxic recovery, gender-identified preBötC slices were exposed to the hypoxia-reoxygenation paradigm in the presence of GABA_A_ and glycinergic receptor blockade using PTX (50 µM) and STR (1 µM). In PTX and STR, f_inst_ was not significantly different for either gender (Figure 8A). Additionally, the gender difference in TTFB was preserved in the presence of PTX and STR (Figure 8B; P = 0.01): TTFB was 384±33 sec and 292±26 sec for male (n = 7) and female (n = 7) rhythmic activities, respectively.

### Postnatal Age Influences the Gender Difference in Post-hypoxic recovery of Respiratory Rhythmic Activity

**Figure 9:**
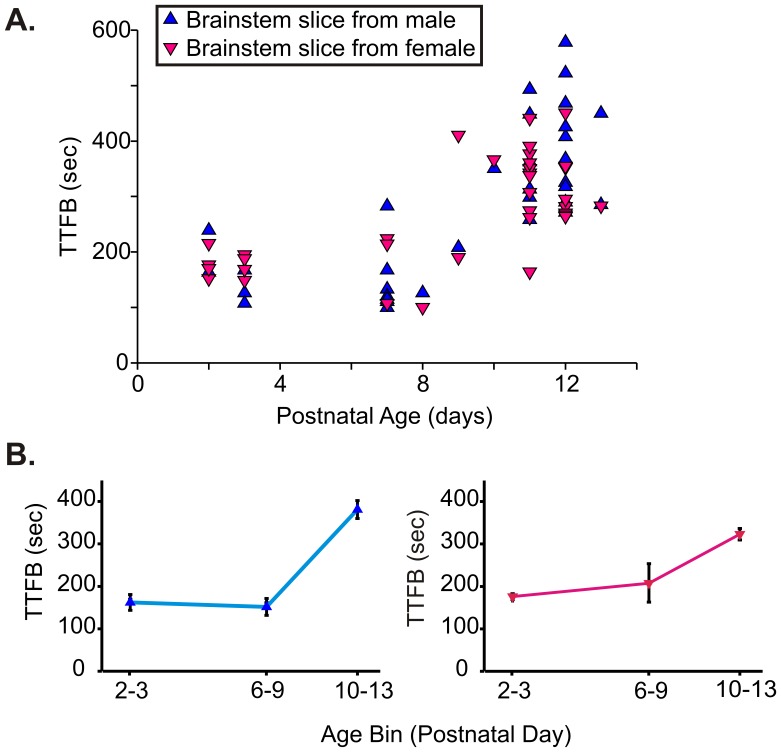
Postnatal age affects post-hypoxic recovery of rhythmogenesis. (A) No gender differences were found in TTFB for age bins at postnatal days 2–3 (male n = 6; female n = 9) or 6–9 (male n = 9, female n = 6). (B) However, TTFB increases with age for both genders between postnatal days 10–13.

To determine whether the gender difference in post-hypoxic recovery was an inherent property to preBötC rhythmic activity, we exposed age matched gender-identified slices to the hypoxia-reoxygenation paradigm. No gender differences were found in the post-hypoxic recovery at the younger age bins (P0–3; P6–9) tested (Figure 9A). However, plotting TTFB following hypoxia as a function of age bin for each gender demonstrated that TTFB increased at the postnatal age bin of P10–13 for both genders (Figure 9B).

## Discussion

Detrimental consequences of hypoxic states caused by central apnea, obstructive apnea, asphyxia, and cardiac arrest are well known. Males show increased vulnerability in many of these conditions [1], [2], [3], [4], [7], [28]. To gain insight into this issue, much attention has been focused on events during hypoxia, yet the acute period of reoxygenation rather than hypoxia itself may also be a critical period of vulnerability for respiratory function. It is well-documented that during reoxygenation and the immediate period following, breathing is centrally depressed [20], [29], [30], [31], [32], [33]. Our study demonstrates that a gender difference exists in the acute recovery of respiration following severe hypoxia. Moreover, we found a similar gender difference preserved in preBötC rhythms recorded from gender-identified brainstem slices.

### Stereotypical Responses of Rhythmogenesis to Hypoxia and Reoxygenation

No differences were found in rhythmogenesis during steady-state hypoxia or in carbogen. However, our in vitro experiments demonstrated that gender differences in in vitro rhythmogenesis not only occurs during post-hypoxic recovery but also during the initial period of hypoxic augmentation. During this period, the frequency of rhythmogenesis is augmented above the mean frequency prior to hypoxia (Figure 3, [20]). While we observed this pattern in all rhythmically active slice preparations, independent of gender, our analysis (Figure 3B) showed that the rhythmic activity in females is more robust during this phase: females had a smaller propensity for early failure in burst generation. Although augmented in vivo respiratory activity during early hypoxia is largely attributed to peripheral sensory input [9], the tendency for failure of preBötC rhythms during this period has potential implications for in vivo gender-biased outcomes during and following insults where peripheral sensory input may be impaired. Moreover, upon reoxygenation, gender differences were apparent in post-hypoxic recovery from rhythmogenesis, both in freely breathing mice and in in vitro preparations.

Unlike the hypoxic augmentation, the post-hypoxic ventilatory depression appears to be primarily of central origin. Although early reports suggested that the ventrolateral pons is required for post-hypoxic ventilatory depression in vivo [29], [30], [31], in vitro studies indicate that the preBötC is a major contributor to the depression as a dramatic post-hypoxic depression is present even in the absence of the pons [19], [20]. Thus, while the post-hypoxic ventilatory depression likely involves several interacting CNS areas, our in vivo and in vitro experiments are consistent in their key findings. While it would be a mistake to conclude that gender differences at the level of the preBötC are the sole basis to our in vivo observations, the in vitro findings clearly indicate that gender differences in post-hypoxic recovery exist at the level of the preBötC and these properties likely contribute to the in vivo gender difference observed at the level of the breathing behavior. Moreover, the analysis of in vitro rhythmogenesis during various periods prior, during, and following hypoxia also indicate that the gender bias in rhythmogenesis is evident only during the dynamic transitions in oxygenation, but not during the steady-state oxygen conditions.

### Mechanistic Gender Differences in Rhythmogenesis

As demonstrated here, rhythmogenesis in males has a greater propensity for early failure during the hypoxic augmentation and the onset of respiratory rhythmic activity is more delayed following reoxygenation. Moreover, the gender bias in TTFB was not present at birth, but developed only after postnatal day 10. When compared to earlier ages, recovery of respiratory activity was significantly increased for both genders at postnatal days 10 to 13. Thus, while TTFB became larger with age, gender differences in post-hypoxic recovery also appear to develop later. In rodents, the second postnatal week is a critical period of development when chloride gradients [34], [35], [36] and GABA_A_ receptor subunit expression changes [37]. However, while such events likely contribute to age dependent changes in post-hypoxic recovery, blockade of fast GABAergic and glycinergic receptors did not prevent the post-hypoxic gender difference in rhythmogenesis. These results suggest that while several biological changes occur during postnatal development, not all appear to equally contribute to gender differences in post-hypoxic recovery of the preBötC rhythm.

In contrast to the role of synaptic inhibition, blockade of K_ATP_ increased f_inst_ of male preBötC rhythms prior to hypoxia, and subsequently eliminated gender differences in the recovery of the preBötC rhythm. Based on the linear correlation found between f_inst_ and TTFB in slices from the male gender, the TOL experiments could be explained by an increasing f_inst_ of individual male rhythms, which is predicted to lead to shorter TFFB values thereby eliminating the gender difference in post-hypoxic recovery. However, an alternative interpretation is that the K_ATP_ activity contributes to the gender differences. While increasing f_inst_ may facilitate the post-hypoxic recovery in rhythmic activity of male slices, the pharmacological experiments best support the alternative interpretation. Specifically, if the gender difference in rhythmogenesis was solely based on differences in male f_inst_, then retarding the f_inst_ of preBötC rhythms should have preserved the gender difference in post-hypoxic recovery since the TTFB in female slices does not correlate to f_inst_. Experiments using diazoxide decreased f_inst_ in rhythms from both genders, yet also eliminated the gender difference in TTFB. Thus, manipulating K_ATP_ activity in either direction appears to eliminate the gender differences in post-hypoxic recovery of rhythmogenesis independent on their effects on instantaneous frequency. The gating of K_ATP_ channels is governed by the ATP/ADP ratio [38], and K_ATP_ provides an avenue by which metabolic status may influence neuronal excitability. While supplementing glucose did not affect preBötC rhythms in male slices, it increased f_inst_ and reduced TTFB in rhythms recorded from female slices. This finding suggests that, at the level of the preBötC, females utilize glucose more efficiently than the male counterparts. Hence, we propose that metabolic status may contribute to the observed gender differences in the post-hypoxic recovery of respiratory rhythmogenesis. This hypothesis is consistent with a potential role for a dynamic involvement of K_ATP_ channels in these responses.

### Potential Consequences

While the prevalence of hypoxic insult and the ensuing reoxygenation are present throughout numerous disease states and conditions, our findings may have particular implications to the development of events leading to SIDS. SIDS has long been associated with the failure to recover from a hypoxic/hypercapnic event, resulting for example, from an infant sleeping in the prone position [39]. Important for autoresuscitation is the activation of gasps in response to severe hypoxic challenge [40], [41]. Mechanistically, gasping depends on the persistent sodium current [19], [42], and is attributed to the activation of sodium dependent pacemaker neurons [19]. Blockade of endogenous serotonergic activation abolishes both gasping and pacemaker activity [32] indicating that gasping and these pacemaker neurons critically depend on serotonergic drive [32], [43]. Consistent with this conclusion is the observation that in serotonin-deficient mice, gasping is markedly attenuated during a short time window in postnatal development [44]. Additionally, recent studies in mice with genetic impairment to serotonergic neuromodulation have demonstrated that males are vulnerable to a loss of chemosensitive responsiveness [45] [46]. However, while both the failure to effectively gasp/autoresuscitate [11] and disturbances in serotonergic mechanisms [47] appear critical in the disease mechanisms of SIDS, other biological factors may also be involved with the etiology of SIDS. Our work suggests that gender differences in acute post-hypoxic recovery can be present even when serotonergic pathways are not purposefully disrupted, but it is likely that such differences may be exaggerated by manipulating metabolic substrate availability or eliminated by altering the K_ATP_ activity(as discussed in the preceding section). Recent findings show that loss-of-function mutations found in Kir6.1, a central subunit to the K_ATP_ channel, are present in some SIDS cases [48]. Moreover, using quantitative trait locus analysis, Thach et al. (2009) recently described gender differences in loci on chromosomes 10 and 12 that appear to be particularly relevant to autoresuscitation. While several genes exist within these loci, the authors identify two particular gene candidates that may contribute to gender differences in autoresuscitation- glycogen synthase kinase-3 and hexokinase-1 [12]. These genes are involved with glycogen/glucose metabolic pathways, and thus, such differences could potentially affect ATP availability during hypoxia/reoxygenation. Thus, our findings also contribute to the growing evidence that implicates a role for differences in metabolic status and K_ATP_ channel activity as a potential source for gender differences during/following hypoxic challenge and SIDS.
